# C-reactive protein levels, the prognostic nutritional index, and the lactate dehydrogenase-to-lymphocyte ratio are important prognostic factors in primary central nervous system lymphoma: a single-center study of 223 patients

**DOI:** 10.1007/s10143-023-02248-1

**Published:** 2023-12-19

**Authors:** Jinyi Zuo, Ting Lei, Shuai Zhong, Jiajun Zhou, Rui Liu, Chenxing Wu, Shouwei Li

**Affiliations:** Department of Neuro-Oncology, Sanbo Brain Hospital, Capital Medical University, Beijing, People’s Republic of China

**Keywords:** Primary central nervous system lymphoma, C-reactive protein, Prognostic nutritional index, Lactate dehydrogenase-to-lymphocyte ratio, Prognostic factor, Elderly patients

## Abstract

Primary central nervous system lymphoma (PCNSL) is a rare and highly aggressive type of extranodal non-Hodgkin lymphoma (NHL), and the prognosis is poor. Currently, the most used prognostic models are the Memorial Sloan-Kettering Cancer Center (MSKCC) and International Extranodal Lymphoma Study Group (IELSG) scores; however, their predictive effects are changing with increasing incidence and changing treatment regimens. A growing body of evidence has demonstrated that inflammatory and nutritional markers are factors that can determine tumor prognosis. Therefore, the aim of this study was to identify and validate novel prognostic factors for PCNSL. Clinical information was collected from 223 patients with PCNSL. Patients younger than 18 years of age were excluded. Progression-free survival (PFS) and overall survival (OS) were used as endpoints, and receiver operating characteristic (ROC) curve analyses were conducted to determine the cutoff values for the inflammatory indicators. Correlations between variables and PFS or OS were assessed using univariate and multivariate analyses, and positive indicators were selected for survival analysis. A prognostic nutritional index (PNI) < 49.38 was associated with worse PFS (*p* = 0.003), and outcomes significantly differed between patients with a PNI ≥ 49.38 and < 49.38 (*p* < 0.001). Age < 60 years (*p* < 0.001) and C-reactive protein (CRP) levels < 3.14 (*p* = 0.001) were associated with better OS. In elderly patients (≥ 60 years), a lactate dehydrogenase-to-lymphocyte ratio (LLR) < 95.69 (*p* = 0.021) was associated with better OS, and the outcome significantly differed between patients with an LLR ≥ 95.69 and LLR < 95.69 (*p* = 0.015). The PNI and CRP levels are prognostic factors for PCNSL, and CRP was the first time shown to be a prognosis factor of PCNSL. In elderly patients with PCNSL, the LLR can predict prognosis.

**Supplementary Information:**

The online version contains supplementary material available at 10.1007/s10143-023-02248-1.

## Introduction

Primary central nervous system lymphoma (PCNSL) is a rare and aggressive cancer within the brain, spinal cord, cerebrospinal fluid, or vitreoretinal space. Most recurrent cases involve these locations, with few recurrence can occur outside of the central nervous system (CNS) [1]. PCNSL was recently classified as a type of “large B-cell lymphoma at an immune-privileged site” in the 2022 edition of the World Health Organization Classification of Haematolymphoid Tumours [2], whereas the International Consensus Classification of Mature Lymphatic Neoplasms considers PCNSL to be a distinct disease entity [3]. PCNSL accounts for approximately 4% of all primary CNS tumors and 4–6% of all extranodal lymphoma cases. The annual incidence rate ranges from 0.3 to 0.6 cases per 100,000 individuals [4]. In terms of demographics, a cohort study reported a median age at diagnosis of 67 years, with a higher incidence in males than in females [5, 6]. However, the incidence has slowly increased over the last 50 years, particularly in older patients [7]. The clinical outcomes of PCNSL are poor, with 5-year overall survival (OS) rates of only 15–30% [8], imposing a heavy economic burden on individual patients and society. Therefore, establishing reliable prognostic prediction to accurately stratify patients and guide clinical decision-making is crucial for improving survival outcomes.

Current prognostic assessments of PCNSL are primarily based on clinical status, and the Memorial Sloan-Kettering Cancer Center (MSKCC) and International Extranodal Lymphoma Study Group (IELSG) scores are two of the most widely used prognostic factors. The former divides patient prognosis into three groups: age ≤ 50, age > 50 and the Karnofsky Performance Scale (KPS) score ≥ 70, and age > 50 and the KPS score < 70. The latter comprises five parameters: age > 60, the Eastern Cooperative Oncology Group Performance Status > 1, elevated lactate dehydrogenase (LDH) level, high cerebrospinal fluid protein, and deep brain lesion. Each parameter is assigned one point, and based on the total score, the prognosis is categorized into three groups: 0–1, 2–3, and 4–5. However, recent advances in radiology and molecular sciences [9] have improved the diagnosis and treatment of PCNSL, and the predictive effects of the MSKCC and IELSG scores vary based on the incidence and treatment modality. Therefore, methods for predicting PCNSL prognosis must be updated and improved accordingly.

The prognostic nutritional index (PNI) is an assessment of nutritional and immune status derived from serum albumin levels and lymphocyte counts [10]. It was first used to assess the nutritional and immune status of patients undergoing gastrointestinal surgery [11] and has been gradually adopted as an prognostic indicator in multiple malignancies, including gynecological tumors and lung cancer, and it is increasingly being used to predict prognosis in other non-tumor diseases, including fractures, heart failure, and brain infarction [12]. Recent studies have confirmed that inflammatory responses drive PCNSL development and progression [13–15]. Thus, inflammatory indicators could serve as prognostic factors, and there may also be a link between nutritional status and disease development in older adults [16]. Therefore, this retrospective study aimed to investigate whether PNI, a nutritional indicator, and other laboratory and clinical factors could be used to predict PCNSL prognosis, including the C-reactive protein (CRP) and lactate dehydrogenase-to-lymphocyte ratio (LLR) levels, an inflammatory marker.

## Methods

### Patients

Data from 223 patients with PCNSL who received treatment from the Sanbo Brain Hospital in China between December 2012 and August 2021 were retrospectively collected. PCNSL diagnosis was based on the WHO Classification of Tumors of the Central Nervous System 2021 [2]. The inclusion criteria were as follows: (1) negative test for human immunodeficiency virus in serum; (2) tumor locations limited to the brain, meninges, spinal cord, and eyes, without systemic involvement; (3) no history of other diagnosed malignancies or prior anticancer treatment; (4) no history of immunosuppression or organ transplantation; and (5) no pretreatment, such as with corticosteroid therapy, before study enrollment. Subsequently, patients younger than 18 years of age were excluded. The study was approved by the Institutional Review Board/Ethics Committee of Sanbo Brain Hospital and adhered to the principles of the Declaration of Helsinki. Informed consent was obtained from all patients.

### Data collection

Following PCNSL diagnosis, the patients’ basic and preoperative clinical characteristics were collected. Basic information included age and sex, and the clinical characteristics included data based on magnetic resonance imaging (MRI) of the brain; sites of tumor invasion; surgical modality (biopsy/resection); pathology diagnosis (based on the Hans algorithm); eye involvement; levels of hemoglobin (Hb, g/L), albumin (ALB, g/L), creatinine (Cr, µmol/L), β2-microglobulin (β2-MG, mg/L), lactate dehydrogenase (LDH, U/L), and CRP (mg/L); cell counts for peripheral blood neutrophils (NEU, × 10^9/L), lymphocytes (LYM, × 10^9/L), mononuclear cells (MONO, × 10^9/L), white blood cells (WBC, × 10^9/L), and platelets (PLT, × 10^9/L); the pre-operative KPS score; the IELSG and MSKCC scores; and postoperative treatment regimens. All laboratory test data were obtained before the patient received surgery. The reference values for laboratory parameters can be found in the Online Resource 1, and all reference values were unchanged over time. From these data, the immune inflammation and prognostic nutritional indices were calculated, including the systemic inflammation response index (SIRI), systemic immune inflammation index (SII), neutrophil-to-lymphocyte ratio (NLR), derived neutrophil-to-lymphocyte ratio (dNLR), LLR, and PNI. The formulas used were as follows: SIRI = NEU count × MONO count/LYM count; SII = PLT count × NEU count/LYM count; NLR = NEU count/LYM count; dNLR = NEU count/(WBC count − NEU count); LLR = LDH/LYM count; PNI = ALB + 5 × LYM count.

### Clinical outcomes

According to the International Working Group Recommendations for Response Criteria of non-Hodgkin lymphoma (NHL) [17], treatment responses were classified as complete response (CR), partial response (PR), stable disease (SD), and progressive disease (PD). OS was defined as the time between diagnosis and death from any cause. Progression-free survival (PFS) was defined as the time between diagnosis and the first event (death from any cause or disease progression).

### Statistical analysis

Baseline characteristics of patients were summarized using descriptive statistics. Receiver operating characteristic (ROC) curve analysis was conducted to determine the optimal cutoff values of the target variables. Survival curves were constructed, and survival rates were calculated using the Kaplan–Meier method. The log-rank test was used to analyze statistically significant differences in survival between subgroups. Variables with statistical significance in the univariate analysis were included in the multivariate analysis using the Cox proportional hazards model. All statistical analyses were performed using Statistical Package for the Social Sciences (SPSS) 26.0 software (IBM Corp., Armonk, NY, USA). *p* ≤ 0.05 was considered statistically significant.

## Results

### Patients’ characteristics

After excluding young patients, 218 patients were included in the statistics. Data from 218 patients with confirmed PCNSL were included in the analysis (Table 1). Biopsies for pathological diagnosis were performed in 142 patients (65.0%), and 76 patients (35.0%) underwent resection for tumor removal. The mean age was 57.5 ± 12.3 years, and the male-to-female ratio was 1.22. The most common pathological type was diffuse large B-cell lymphoma (*n* = 205, 94.0%); of these patients, 43 (19.7%) had the germinal center B cell–like (GCB) type, whereas 162 (74.3%) had the nongerminal center B cell–like (non-GCB) type. The characteristics of patients diagnosed with DLBCL are summarized in online resource 2. Among the 13 non-DLBCL patients, three were diagnosed with extranodal marginal zone B-cell lymphoma of mucosa-associated lymphoid tissue (MALT lymphoma), three with Burkitt lymphoma, and seven with mature T and/or NK cell lymphoma. Deep brain structure invasion was observed in 176 patients (80.7%). None of the patients was found to have eye involvement.

**Table 1:** Characteristics of 218 patients with PCNSL

Characteristics	Total
	*n* = 218
Surgical modality, *n*%	
Biopsy	142, 65.0%
Resection	76, 35.0%
Age, years	
Mean ± standard deviation	57.5 ± 12.3
Median	58
Range	22–86
Gender, *n*%	
Male	120, 55.0%
Female	98, 45.0%
Deep brain lesion, *n*%	
Yes	176, 80.7%
No	42, 19.3%
Pathology, *n*%	
GCB	43, 19.7%
Non-GC	162, 74.3%
Other	13, 6.0%
MRI T1 (*n* = 177), *n*%	
Isointense	8, 4.5%
Hypointense	166, 93.8%
Hyperintense	3, 1.7%
MRI T2 (*n* = 177), *n*%	
Isointense	10, 5.6%
Hypointense	7, 4.0%
Hyperintense	160, 90.4%
Preoperative KPS, *n*%	
≥ 70	175, 80.3%
< 70	43, 19.7%
Treatment (*n* = 170), *n*%	
CMT	139, 81.8%
CMT + WBRT	26, 15.3%
WBRT	5, 2.9%
CMT type (*n* = 165), *n*%	
MTX	139, 84.2%
Other	26, 15.8%

*GCB* germinal center B cell-like, *non-GCB* nongerminal center B cell-like, *KPS* Karnofsky performance score, *CMT* chemotherapy, *WBRT* whole brain radiotherapy, *MTX* methotrexate

MRI data were available for 177 patients (81.2%). In the T1-weighted imaging, hypointensity was observed for 166 (93.8%) patients; only 8 (4.5%) and 3 (1.7%) patients exhibited isointensity and hyperintensity, respectively, relative to that of the gray matter. In the T2-weighted imaging, 160 (90.7%) patients exhibited hyperintensity; only 10 (5.6%) and 7 (4.0%) patients exhibited isointensity and hypointensity, respectively. There were 175 (80.3%) and 43 (19.7%) patients with KPS scores ≥ 70 and < 70, respectively.

### Treatment and responses

Following biopsy or resection, 170 patients (78.0%) underwent treatment. There are 48 patients (22.0%) who did not receive treatment. Among them, 30 patients were missing treatment data due to loss to follow-up, 9 patients declined chemotherapy due to intolerance and advanced age (with an average age of 80 years), and the remaining 9 patients discontinued subsequent treatment due to disease progression before initiating therapy. Among the 170 patients, 165 (97.1%) received chemotherapy (CMT), with 26 (15.3%) of them also undergoing whole brain radiotherapy (WBRT), while an additional 5 patients (2.9%) received WBRT alone. Among all patients received CMT, most common regimen was high-dose methotrexate (HD-MTX; *n* = 139, 84.2%)-based treatment, including methotrexate monotherapy (M; *n* = 10, 7.2%), methotrexate + temozolomide (MT; *n* = 114, 82.0%), methotrexate + temozolomide + pemetrexed (MTP; *n* = 8, 5.8%), methotrexate + temozolomide + etoposide (MTE; *n* = 6, 4.3%), and methotrexate + cytarabine (MA, *n* = 1, 0.7%). Other CMT regimens included etoposide + temozolomide (*n* = 19, 11.5%) and pemetrexed monotherapy (P; *n* = 1, 0.6%). Seventeen (10.3%) and 10 (6.1%) patients also received rituximab (R) and lenalidomide, respectively. Bruton’s tyrosine kinase (BTK) inhibitors and programmed cell death protein 1 (PD-1) were administered to five (3.0%) and two (1.2%) patients, respectively. After initial therapy, 54 patients (31.8%) achieved CR, and 14 (8.2%) achieved PR; 89 (52.4%) and 9 (5.3%) patients were classified as PD and SD, respectively. Data on treatment responses were unavailable for 4 (2.4%) patients.

### Determination of cutoff values

**Fig. 1:**
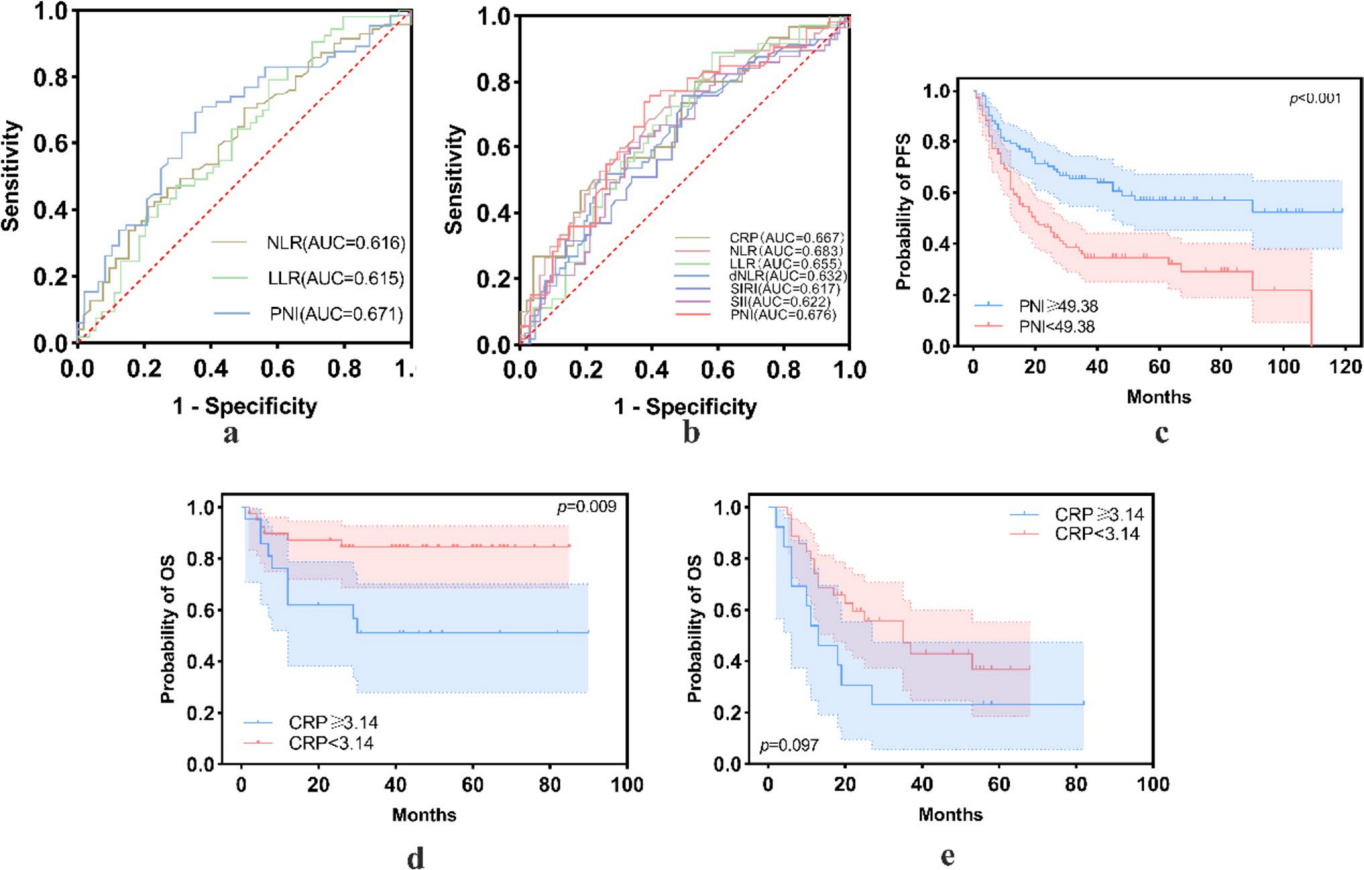
ROC curves and Kaplan–Meier survival curves of PFS and OS. NLR (*p* = 0.024), LLR (*p* = 0.035), and PNI (*p* = 0.001) were statistically significant on the ROC curve of PFS (**a**). CRP level (*p* = 0.008), NLR (*p* < 0.001), LLR (*p* = 0.004), dNLR (*p* = 0.005), SIRI (*p* = 0.022), SII (*p* = 0.017), and PNI (*p* = 0.001) were statistically significant on ROC curve of OS (**b**). Kaplan–Meier survival curves of PFS demonstrated that PNI was associated with PFS (*p* < 0.001) (**c**). In patients younger than 60 years, those with a CRP level < 3.14 mg/L exhibited better OS than those with a CRP level ≥ 3.14 mg/L (*p* = 0.009) (**d**). While no significant difference was observed between the two groups in patients aged 60 years or older (*p* = 0.097) (**e**)

OS and PFS were used as endpoints for the ROC analysis, which was conducted to calculate the optimal cutoff values for CRP, NLR, LLR, dNLR, SIRI, SII, PNI, and β2-MG. When PFS was used as the endpoint, the NLR (*p* = 0.024), LLR (*p* = 0.035), and PNI (*p* = 0.001) were statistically significant. The area under the curve (AUC) values for the NLR, LLR, and PNI were 0.616, 0.615, and 0.671, respectively, with respective cutoff values of 2.34, 95.95, and 49.38. With OS as the endpoint, the CRP level (*p* = 0.008), NLR (*p* < 0.001), LLR (*p* = 0.004), dNLR (*p* = 0.005), SIRI (*p* = 0.022), SII (*p* = 0.017), and PNI (*p* = 0.001) were statistically significant, with respective AUC values of 0.667, 0.683, 0.655, 0.632, 0.617, 0.622, and 0.676 and respective cutoff values of 3.14, 2.58, 95.95, 2.37, 0.82, 518.21, and 49.63. The ROC curves are shown in Fig. 1.

### Survival and prognosis

Of the 218 patients, 184 (86.2%) had complete follow-up data; 34 patients were lost to follow-up due to refusal to continue or a change in contact information. The median follow-up time for patients lost to follow-up was 58 months. In the univariate analysis (Table 2 and 3), age ≥ 60 years, PLT count, ALB level, being classified in the intermediate-risk group based on the IESLG score or the intermediate-risk or high-risk group based on the MSKCC score, NLR ≥ 2.34, LLR ≥ 95.95, and PNI ≥ 49.38 were factors that significantly affected PFS. OS was significantly affected by an age ≥ 60 years, PLT count, ALB level, pathological type, pre-operative KPS score ≥ 70, IELSG score, MSKCC score, treatment type, CRP level ≥ 3.14, NLR ≥ 2.58, LLR ≥ 95.95, dNLR ≥ 2.37, SII ≥ 518.21, and PNI ≥ 49.63.

**Table 2:** Univariate analysis of continuous variables associated with PFS and OS

Continuous variable	Data available (%)	Average	FPS			OS		
			HR	95%CI	*p*	HR	95%CI	*p*
Operation time (min)	95	104.15	0.999	0.997–1.001	0.387	0.999	0.997–1.001	0.190
Hemoglobin (g/L)	94	135.20	0.995	0.984–1.007	0.404	0.995	0.983–1.008	0.478
WBC (× 10^9/L)	94	7.1	0.996	0.920–1.080	0.931	1.002	0.916–1.097	0.961
PLT (× 10^9)	94	206.54	0.995	0.992–0.999	0.006*	0.995	0.991–0.999	0.007*
ALB (g/L)	88	40.17	0.953	0.913–0.995	0.030*	0.947	0.903–0.993	0.025*
Cr (µmol/L)	89	63.02	1.008	0.995–1.020	0.228	1.004	0.990–1.018	0.573
β2-MG (mg/L)	73	0.21	0.328	0.059–1.822	0.203	0.354	0.052–2.403	0.288
NEU (× 10^9/L)	94	5.17	1.012	0.989–1.035	0.307	1.017	0.995–1.040	0.135
LYM (× 10^9/L)	94	1.76	0.908	0.712–1.158	0.436	0.901	0.682–1.192	0.466
MONO (× 10^9/L)	94	0.50	0.644	0.325–1.276	0.208	0.442	0.159–1.227	0.117
LDH (U/L)	66	194.20	1.000	0.997–1.003	0.926	1.001	0.998–1.004	0.470

^*^p value < 0.05; **p value < 0.001

*WBC* white blood cells, *PLT* platelets, *ALB* albumin, *Cr* creatinine, *β2-MG* β2-microglobulin, *NEU* peripheral blood neutrophil count, *LYM* lymphocyte count, *MONO* mononuclear cell count, *LDH* lactate dehydrogenase, *HR* hazard ratio, *CI* confidence interval

**Table 3:** Univariate analysis of categorical variables associated with PFS and OS

Categorical variable	*N*	%	PFS			OS		
			HR	95%CI	*p*	HR	95%CI	*p*
Surgical modality Biopsy Resection								
	142	65						
	76	35	0.979	0.671–1.428	0.912	0.906	0.593–1.384	0.648
Age ≥ 60 Yes No								
	101	46						
	117	54	0.418	0.288–0.607	< 0.001**	0.321	0.210–0.493	< 0.001**
Pathology GCB Non-GCB Other								
	43	20						
	162	74	1.566	0.933–2.627	0.089	1.972	1.049–3.708	0.035*
	13	6	1.810	0.780–4.198	0.167	2.598	1.005–6.714	0.049*
KPS ≥ 70 Yes No								
	175	80						
	43	20	1.442	0.931–2.232	0.101	1.831	1.149–2.918	0.011*
Treatment								
CMT	139	64						
CMT + WBRT	26	12	0.800	0.453–1.413	0.442	0.621	0.318–1.212	0.163
WBRT	5	2	2.013	0.812–4.992	0.131	2.538	1.015–6.346	0.046*
CMT type								
MTX	139	64						
Other	26	12	0.823	0.448–1.510	0.529	1.123	0.589–2.142	0.724
IELSG Low Intermediate High								
	103	47						
	110	50	1.530	1.053–2.224	0.026*	1.826	1.200–2.777	0.005*
	3	1	3.357	0.807–13.957	0.096	4.672	1.108–19.703	0.036*
MSKCC Low Intermediate High								
	55	25						
	130	60	2.336	1.394–3.915	0.001*	2.352	1.311–4.220	0.004*
	32	15	3.148	1.649–6.011	0.001*	4.668	2.109–8.690	< 0.001*
CRP ≥ 3.14 Yes No								
	34	16	-	-	-			
	74	34	-	-	-	0.462	0.256–0.833	0.010*
NLR ≥ 2.34 Yes No								
	120	55				-	-	-
	85	39	0.641	0.435–0.945	0.025*	-	-	-
NLR ≥ 2.58 Yes No								
	106	49	-	-	-			
	99	45	-	-	-	0.559	0.366–0.854	0.007*
LLR ≥ 95.95 Yes No								
	86	39						
	57	26	0.553	0.338–0.905	0.018*	0.386	0.207–0.720	0.003*
dNLR ≥ 2.37 Yes No								
	67	31	-	-	-			
	138	63	-	-	-	0.494	0.325–0.749	0.001*
SIRI ≥ 0.82 Yes No								
	127	58	-	-	-			
	78	36	-	-	-	0.703	0.453–1.091	0.116
SII ≥ 518.21 Yes No								
	104	48	-	-	-			
	101	46	-	-	-	0.656	0.431–0.999	0.049*
PNI ≥ 49.38 Yes No								
	92	42				-	-	-
	100	46	2.235	1.493–3.345	< 0.001**	-	-	-
PNI ≥ 49.63 Yes No								
	89	41	-	-	-			
	103	47	-	-	-	2.654	1.664–4.235	< 0.001**

^*^p value < 0.05; **p value < 0.001

*GCB* germinal center B cell-like, *non-GCB* nongerminal center B cell-like, *KPS* Karnofsky performance score, *CMT* chemotherapy, *WBRT* whole brain radiotherapy, *MTX* methotrexate, *IELSG* International Extranodal Lymphoma Study Group, *MSKCC* Memorial Sloan Kettering Cancer Center model, *CRP* C-reactive protein, *NLR* neutrophil-to-lymphocyte ratio, *LLR* lactate dehydrogenase-to-lymphocyte ratio, *dNLR* derived neutrophil-to-lymphocyte ratio, *SIRI* systemic inflammation response index, *SII* systemic immune inflammation index, *PNI* prognostic nutritional index, *HR* hazard ratio, *CI* confidence interval

In the multivariate analysis for PFS, a PNI < 49.38 was associated with worse PFS (*p* = 0.003; hazard ratio [HR] = 2.066; 95% confidence interval [CI], 1.28–3.35), after controlling for age, PLT count, ALB level, IESLG score, MSKCC score, NLR, and LLR (Table 4). PNI data were available for 192 (88.1%) patients. A survival curve was constructed to plot the relationship between PNI and PFS (Fig. 1), and the log-rank test revealed significant differences (*p* < 0.001) depending on the PNI; patients with a PNI ≥ 49.38 had a mean PFS of 74.9 months; those with a PNI < 49.38 had a mean PFS of just 42.2 months. In the multivariate analysis for OS, after controlling for the PLT count, ALB level, pathological type, pre-operative KPS score, IELSG score, MSKCC score, treatment type, NLR, LLR, dNLR, SII, and PNI, an age < 60 years (*p* < 0.001; HR = 0.25; 95%CI, 0.12–0.54) and a CRP level < 3.14 mg/L (*p* = 0.001; HR = 0.28; 95%CI, 0.13–0.58) were associated with better OS (Table 4).

**Table 4:** Multivariate analysis of variables associated with PFS and OS

Variable	PFS			OS		
	HR	95%CI	*p* value	HR	95%CI	*p* value
PNI ≥ 49.38 (PFS) Yes No				N/A		
	2.066	1.276–3.345	0.003*			
Age ≥ 60 Yes No	N/A					
				0.250	0.116–0.537	< 0.001**
CRP ≥ 3.14 (OS) Yes No	N/A					
				0.275	0.130–0.579	0.001*

^**^p value < 0.001; *p value < 0.05

*PNI* prognostic nutritional index, *CRP* C-reactive protein, *N/A* not available, *HR* hazard ratio, *CI* confidence interval

Sufficient CRP level data were available for 109 patients (50.0%). We investigated whether the patients could be stratified according to age based on a CRP ≥ 3.14 mg/L and < 3.14 mg/L. In patients younger than 60 years, those with a CRP level < 3.14 mg/L exhibited better OS than those with a CRP level ≥ 3.14 mg/L (*p* = 0.009); no significant difference was observed between the two groups in patients aged 60 years or older (*p* = 0.097) (Fig. 1).

### Further stratification of older patients

**Fig. 2:**
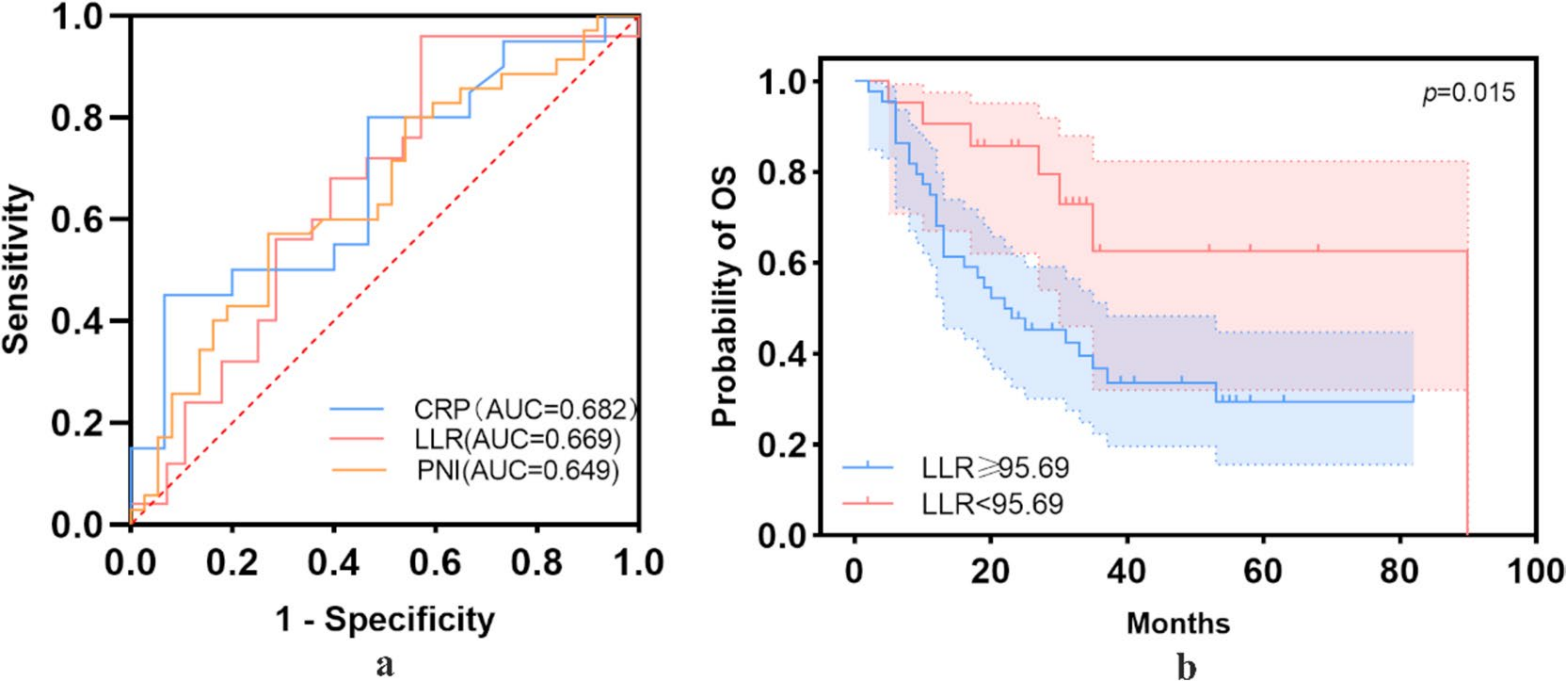
ROC curve and Kaplan–Meier survival curve of OS in elderly patients (age ≥ 60 years). CRP level (*p* = 0.047), LLR (*p* = 0.027), and PNI (*p* = 0.022) were statistically significant on ROC curve of OS (**a**). Kaplan–Meier survival curves of OS demonstrated that LLR was associated with OS in elderly patients (*p* = 0.015) (**b**)

Univariate and multivariate Cox regression analyses were performed in elderly patients (age ≥ 60 years; *n* = 101, 45.3%) to identify variables that independently influenced survival outcomes. With OS as the endpoint, ROC curve analysis was performed to calculate the cutoff values for the CRP level, NLR, LLR, dNLR, SIRI, SII, PNI, and β2-MG level, which revealed that the CRP level (*p* = 0.047), LLR (*p* = 0.027), and PNI (*p* = 0.022) were statistically significant. The AUC values of the CRP, LLR, and PNI were 0.682, 0.669, and 0.649, respectively, and the cutoff values were 2.8, 95.69, and 46.23, respectively (Fig. 2). In the univariate analysis of OS, age, and deep brain lesion tumors, an LLR ≥ 95.69 and PNI ≥ 46.23 were statistically significant (Table 5). In the multivariable analysis of OS, after controlling for age, deep brain lesion tumors, and PNI ≥ 46.23, an LLR < 95.69 was associated with better OS (*p* = 0.021; HR = 0.36; 95%CI, 0.15–0.86) (Table 5). The log-rank test revealed a statistical difference between elderly patients with an LLR ≥ 95.69 and LLR < 95.69 (*p* = 0.015, Fig. 2).

**Table 5:** Univariate and multivariate analysis of PCNSL patients aged ≥ 60 years

Variable (univariate analysis)	%	Median	OS		
			HR	95%CI	*p*
**Age**	100	67.74	1.055	1.018–1.093	0.003*
**Deep brain lesion tumors** **Yes** **No**					
	80				
	20	N/A	1.786	1.014–3.143	0.044*
**LLR ≥ 95.69** **Yes** **No**					
	44				
	21	N/A	0.355	0.147–0.858	0.021*
**Variable (multivariate analysis)**	**%**	**Median**	**OS**		
			**HR**	**95%CI**	***p***
**LLR ≥ 95.69** **Yes** **No**					
	44				
	21	N/A	0.355	0.174–0.858	0.021*

^*^p value < 0.05

*LLR* lactate dehydrogenase-to-lymphocyte ratio, *HR* hazard ratio, *CI* confidence interval

## Discussion

PCNSL is a rare CNS malignancy whose incidence continues to rise, particularly among the elderly. Primary treatments include HD-MTX-based CMT, WBRT, and surgical resection; however, no standardized treatment regimen exists. Recently, molecularly targeted drugs, autologous hematopoietic stem cell transplantation, chimeric antigen receptor T-cell immunotherapy, and other therapeutic modalities have been added to treatment regimens. The most common treatment modality in this study population was HD-MTX-based CMT (*n* = 139, 84.2%), and some patients received targeted therapy with lenalidomide and BTK inhibitors. Most patients were classified with PD (*n* = 89, 52.4%); in addition, 54 patients (31.8%) achieved CR, 14 (8.2%) achieved PR, and 9 patients (5.3%) achieved SD; these results confirm that reliable prognostic factors must be identified to optimize treatment regimens and cure rates.

A variety of nutritional and inflammatory parameters were evaluated, including WBC, PLT, NEU, LYM, and MONO counts; Hb, ALB, CR, LDH, CRP, and β2-MG levels; NLR, LLR, dNLR, SIRI, SII, PNI, and IELSG; and MSKCC scores. The univariate analysis revealed that age, PLT count, ALB level, an intermediate-risk IESLG score, intermediate-risk and high-risk MSKCC scores, NLR, LLR, and PNI were significantly associated with PFS and OS was associated with age, PLT count, ALB and CRP levels, pathological type, preoperative KPS score, IELSG score, MSKCC score, treatment type, NLR, LLR, dNLR, SII, and PNI. The multivariate analysis revealed that PNI was a significant predictor of PFS, whereas age and CRP levels were significant predictors of OS. In those ≥ 60 years, LLR significantly affected OS in the multivariate analysis. The cutoff values of the positive variables were used to stratify patients, and the differences in prognosis between the groups were significant.

The involvement of inflammation in tumor progression has recently become a focal point of oncology research, as inflammation is involved in tumor growth, including shaping the microenvironment and promoting cancer cell proliferation, survival, and migration [18]. Many inflammatory parameters correlate with lymphoma prognosis, including the NLR [14], dNLR [19], lymphocyte-to-monocyte ratio [20], and SIRI [21]; however, none of these was significant associated with survival outcomes in this study.

The relationship between CRP levels and NHL prognosis has been extensively investigated as far back as the pre-rituximab era [22]. More recent studies have confirmed that CRP levels can predict diffuse large B-cell lymphoma (DLBCL) prognosis, with Troppan et al. [23] reporting a strong association between elevated CRP levels and 5-year OS. This study has discovered for the first time an association between CRP and PCNSL, which were no prior foundational or clinical research findings in this area and providing a direction for further exploration of the biological relationship between these two.

Lactate dehydrogenase A (LDH-A) is the key enzyme in the Warburg’s effect, suggesting that tumor cells are more likely to undergo glycolysis than oxidative phosphorylation, despite existing in an aerobic environment. LDH has the highest prognostic impact among the International Prognostic Indices (IPI) [24]. Moreover, targeting LDH-A could represent a potential cancer treatment [25], as LDH plays a role in metastatic colorectal cancer [26]. The poor prognosis of malignant hematological diseases is likely associated with elevated serum LDH levels [27]. The LLR has been used to re-stratify MSKCC scores to assess OS in patients with PCNSL who underwent CMT alone, with significant differences observed between the new subgroups [15]. In this study, LLR was used as a stratification criterion in the elderly patients, revealing significant differences in OS between groups.

The PNI is used to assess the nutritional status of patients undergoing surgery, predict surgical risks, and make prognostic judgments. As mentioned, it is calculated based on levels of albumin and lymphocyte count. Lymphocytes have been found to play a role in anti-tumor immunity, and dysfunction or imbalance in lymphocyte function is associated with tumor progression [10, 28, 29]. Recently, lymphocyte count has been identified as a prognostic factor in extranodal natural killer/T-cell lymphoma [30]. Serum albumin has consistently been used as an indicator of malnutrition, and hypoalbuminemia is associated with immune dysfunction and systemic inflammation [31]. Biologically, albumin levels are considered closely linked to drug biodistribution and bioavailability [32, 33]. Low albumin levels favor the development of tumors and the inflammatory environment associated with cancer, leading to a poorer prognosis [29]. Therefore, it is reasonable that PNI might have considerable prognostic value in PCNSL patients. Tejpal et al. [34] found that the mean PNI significantly differed between patients with PCNSL (*n* = 42) and high-grade glioma (*n* = 16). A retrospective PCNSL study reported an association between the PNI and OS in the univariate analysis (*p* = 0.004) but not the multivariate analysis [21]. Wong et al. [29] found that the PNI impact both OS and PFS in a retrospective experiment of 41 PCNSL patients. In the present study, the PNI correlated with both PFS and OS in the univariate analysis, whereas it was only significantly correlated with PFS in the multivariate analysis.

The association between poorer OS in PCNSL and an age ≥ 60 years was first reported in the twentieth century [35, 36], and age remains one of the most important prognostic parameters in PCNSL. A recent retrospective study reported poorer outcomes in older patients with PCNSL, with a median PFS of 17 months and a median OS of 34 months [7]. The multivariate analysis revealed that poorer PFS was associated with older age at diagnosis and worse Eastern Cooperative Oncology Group Performance Score (ECOG PS), whereas OS was associated with an older age at diagnosis, hypoalbuminemia, higher Cumulative Illness Rating Scale-Geriatric score, and a worse ECOG PS. Moreover, after controlling for other positive variables, this study compared OS between groups following age stratification, which demonstrated that the oldest age group (≥ 60 years) had the lowest survival rate. Pre-treatment assessment of elderly patients with lymphoma can improve treatment planning to optimize survival outcomes [37], and the PCNSL prognostic model produced by Liu et al. also included age ≥ 60 years in the scoring system [38]. This study also assessed other variables that are known to affect the quality of life of older individuals, such as HB, ALB, and Cr levels, which ultimately led to the identification of LLR as a factor associated with OS in elderly patients with PCNSL in the multivariate analysis.

The IELSG and MSKCC score, wildly utilized in clinical practice, each have their own limitations. For example, the IELSG score was shown to have poor separation in terms of PFS and OS, and the MSKCC score had insufficient discriminative ability for PFS in a training cohort [38]. Additionally, the non-compliance of patients, unnecessary examinations, and contraindications to lumbar punctures all contribute to difficulties in obtaining cerebrospinal fluid protein results. Therefore, it is difficult to predict the prognostic by IELSG effectively. And for MSKCC, relying solely on age and KPS for risk stratification still presents biases, potentially resulting in a weaker association between MSKCC and the prognosis of PCNSL. Previous studies have demonstrated this observation [39, 40]. All mentioned above highlight the need for novel PCNSL prediction models. Three trials on 174, 195, and 248 PCNSL patients modeled prognosis with SII, serum albumin, and LLR, respectively [15, 41, 42]. Furthermore, this study also explored pre-treatment parameters affecting both PFS and OS, ultimately leading to the identification of CRP levels and age as prognostic factors in PCNSL. Additionally, elderly patients with PCNSL were stratified according to the LLR, which revealed significant differences in survival outcomes between groups.

Some limitations of this study include the inevitable selection bias inherent to retrospective studies and the lack of a validation cohort. It was also impossible for physicians to prospectively collect patient assessment information before treatment. At the same time, although patients did not exhibit symptoms of infection before undergoing treatment, the potential for undetected infections and autoimmune issues in some patients remains unavoidable. These issues may affect the true relationship between inflammatory markers and the prognosis of PCNSL. Subsequent studies should take further measures to avoid the presence of such conditions.

## Conclusion

The PNI and CRP levels were prognostic factors in patients with PCNSL, and this study identified the significance of the LLR among elderly patients with PCNSL. These three easily evaluated parameters have potential prognostic value, which should be further validated in prospective studies.

## Supplementary Information

Below is the link to the electronic supplementary material.

Supplementary file1 (PDF 141 KB)

Supplementary file2 (PDF 138 KB)

## Data Availability

The data that support the findings of this study are not openly available due to reasons of sensitivity and are available from the corresponding author upon reasonable request.
