# Supplementary material for: C-reactive protein levels, the prognostic nutritional index, and the lactate dehydrogenase-to-lymphocyte ratio are important prognostic factors in primary central nervous system lymphoma: a single-center study of 223 patients
Source: Neurosurg Rev. 2023 Dec 19;47(1):17. doi: 10.1007/s10143-023-02248-1 (PMC10730673; doi:10.1007/s10143-023-02248-1)
Supplement: Supplementary file 1 — Supplementary file1 (PDF 141 KB) [file 10143_2023_2248_MOESM1_ESM.pdf]

Article title: *C-reactive protein levels, the prognostic nutritional index, and the lactate dehydrogenase-to-lymphocyte ratio are important prognostic factors in primary central nervous system lymphoma: A single-center study of 223 patients.*

Journal name: Neurosurgical Review

Author names: Jinyi Zuo[1] , Ting Lei[1] , Shuai Zhong[1] , Jiajun Zhou[1] , Rui Liu[1] , Chenxing Wu[1] , Shouwei Li[1*]

Affiliation: [1]Department of Neuro-oncology, Capital Medical University Sanbo Brain Hospital, Beijing, People's Republic of China

[*] Corresponding author: Shouwei Li

Email: lishouwei@ccmu.edu.cn

Online Resource 1: The reference values for laboratory parameters

Laboratory Parameters	Reference Values
**Hemoglobin(g/L)**	110-160
**WBC(x10^9/L)**	4-10
**PLT(x10^9)**	100-350
**ALB(g/L)**	35-52
**Cr(umol/L)**	45-84
**β2-MG(mg/L)**	0.10-0.30
**NEU(x10^9/L)**	2.00-7.00
**LYM(x10^9/L)**	0.80-4.00
**MONO(x10^9/L)**	0.12-0.80
**LDH(U/L)**	25-247
**CRP(mg/L)**	0-6

WBC: white blood cells; PLT: platelets; ALB: albumin; Cr: creatinine; β2-MG: β2-microglobulin; NEU: peripheral blood neutrophil count; LYM: lymphocyte count; MONO: mononuclear cell count; LDH: lactate dehydrogenase; CRP: C-reactive protein
