# Supplementary material for: C-reactive protein levels, the prognostic nutritional index, and the lactate dehydrogenase-to-lymphocyte ratio are important prognostic factors in primary central nervous system lymphoma: a single-center study of 223 patients
Source: Neurosurg Rev. 2023 Dec 19;47(1):17. doi: 10.1007/s10143-023-02248-1 (PMC10730673; doi:10.1007/s10143-023-02248-1)
Supplement: Supplementary file 2 — Supplementary file2 (PDF 138 KB) [file 10143_2023_2248_MOESM2_ESM.pdf]

Article title: *C-reactive protein levels, the prognostic nutritional index, and the lactate dehydrogenase-to-lymphocyte ratio are important prognostic factors in primary central nervous system lymphoma: A single-center study of 223 patients.*

Journal name: Neurosurgical Review

Author names: Jinyi Zuo[1] , Ting Lei[1] , Shuai Zhong[1] , Jiajun Zhou[1] , Rui Liu[1] , Chenxing Wu[1] , Shouwei Li[1*]

Affiliation: [1]Department of Neuro-oncology, Capital Medical University Sanbo Brain Hospital, Beijing, People's Republic of China

[*] Corresponding author: Shouwei Li

Email: lishouwei@ccmu.edu.cn

Online Resource 2: Characteristics of patients diagnosed with DLBCL.

**Characteristics**	**Total**
	**n=205**
Surgical Modality, n%	
Biopsy	135 65.9%
Resection	70 34.1%
Age, years	
Mean ± standard deviation	57.8±12.0
Median	59
Range	22-85
Gender, n%	
Male	111 54.1%
Female	94 45.9%
Deep brain lesion, n%	
Yes	167 81.5%
No	38 18.5%
Pathology, n%	
GCB	43 21.0%
Non-GC	162 79.0%
MRI T1(n=168) , n%	
Isointense	7 4.2%
Hypointense	158 94.0%

Hyperintense	3 1.2%
MRI T2(n=168) , n%	
Isointense	10 6.0%
Hypointense	2 1.2%
Hyperintense	156 92.8%
Preoperative KPS, n%	
≥70	164 80.0%
< 70	41 20.0%
Treatment(n=164), n%	
CMT	135 82.3%
CMT+WBRT	25 15.2%
WBRT	4 2.5%
CMT type(n=160), n%	
MTX	135 84.3%
Other	25 15.7%

DLBCL: Diffuse Large B-cell Lymphoma; GCB: germinal center B cell-like; non-GCB: nongerminal center B cell-like; KPS: Karnofsky performance score; CMT: Chemotherapy; WBRT: Whole brain radiotherapy; MTX: Methotrexate.
